# Efficacy of *Jia Wei Yang He* formula as an adjunctive therapy for asthma: study protocol for a randomized, double blinded, controlled trial

**DOI:** 10.1186/s13063-018-2739-8

**Published:** 2018-07-04

**Authors:** Wenhan Jiang, Zifeng Ma, Huiyong Zhang, Henry S. Lynn, Beiqi Xu, Xiao Zhang, Rongrong Bi, Jiyou Fu, Yue Chen, Zhen Xiao, Zhijie Zhang, Zhenhui Lu

**Affiliations:** 1 Department of Biostatistics, School of Public Health, Fudan University, Shanghai, People’s Republic of China; 2 Department of Epidemiology, School of Public Health, Fudan University, 130 Dongan Road, No.8 building, Xuhui District, Shanghai, People’s Republic of China; 3 Department of Respiratory, Longhua Hospital Shanghai University of Traditional Chinese Medicine, No.725 South Wanping Road, No.1 building, Xuhui District, Shanghai, People’s Republic of China; 4 School of Epidemiology, Public Health and Preventive Medicine, Faculty of Medicine, University of Ottawa, Ottawa, ON Canada

## Abstract

**Background:**

Over the past two or three decades, the prevalence of asthma has significantly increased worldwide; therefore, effective treatment without side effects is of utmost importance. Traditional Chinese medicine (TCM) plays a vital role in reducing symptoms and improving the quality of life in persistent-asthma patients. The aim of this study is to evaluate the efficacy of the *Jia Wei Yang He* (JWYH) formula in the treatment of asthma and to explore the relationship between the airway microbiome and TCM treatment in asthma patients.

**Methods/design:**

This multicenter, parallel-arm, randomized, double-blinded, placebo-controlled trial will assess the efficacy of JWYH in asthma patients with usual care. Persistent-asthma patients without life-threatening disease will be enrolled on a random basis and are equally assigned to a high- or a low-dose JWYH plus usual care group, or a placebo plus usual care group. Patients are followed up for 4 months. Accordingly, 240 patients will yield sufficient statistical power to determine a difference between groups. Based on modified intent-to-treat (mITT) analyses, the three groups will be compared at 4 weeks after the beginning of treatment. The primary efficacy measurement is the mean change in the Asthma Control Test (ACT) score from baseline to 4 weeks post treatment. Secondary outcomes include forced expiratory volume in 1 s (FEV_1_), forced vital capacity (FVC), peak expiratory flow (PEF), and asthma exacerbations.

This trial also includes analyses of the associations between airway microbiome and asthma treatment.

**Discussion:**

In this study, a randomized clinical trial design is described. The results are based on several outcomes that estimate the efficacy of the JWYH formula and prospective links between the airway microbiome and asthma treatment.

**Trial registration:**

ClinicalTrials.gov, ID: NCT03299322. Registered on 3 October 2017.

**Electronic supplementary material:**

The online version of this article (10.1186/s13063-018-2739-8) contains supplementary material, which is available to authorized users.

## Background

Asthma is a chronic airway inflammatory disease, symptoms of which include recurrent episodes of wheezing, breathlessness, chest tightness, and coughing [1]. In general, these symptoms are associated with airway hyperactivity and accompanied with variable and often reversible airflow obstruction [1]. In recent decades, the incidence of asthma has been significantly increased, and is estimated to affect 300 million individuals worldwide, including both adults and children [2]. According to the guidelines for managing asthma in China, the prevalence of asthma in the adult population is 1.24%, and during the past 30 years, the prevalence has increased [3]. The rising incidence of asthma in the developed world indicates that the control and treatment of asthma is still inadequate; therefore, the current treatment regimen needs to be further improved [4, 5].

As recommended by the Global Initiative on Asthma (GINA) guidelines, inhaled corticosteroids (ICS) and long-acting β_2_-adrenoceptor agonists are the treatment of choice for most patients with asthma [1]. Several patients may benefit from these treatments; however, the condition of many patients is not effectively controlled and their quality of life has not improved. In addition, several patients experience exacerbations because they do not tolerate treatment [6–8] or side effects, such as adrenal suppression, anaphylaxis, and osteoporosis that have been reported from long-term use of the above-mentioned medications [9–12].

Due to the chronic nature of asthma combined with the lack of satisfying and curative treatment, many asthma patients seek traditional Chinese medicine (TCM) as a complementary and alternative medicine (CAM) treatment [13, 14]. A previous study shows that the use of CAM in asthma patients ranged from 4 to 79% [15]. Since 2005, several double-blind, placebo-controlled clinical studies have demonstrated the efficacy and safety of herbal medicines for treating asthma [13, 14, 16, 17]. Data from these studies show that herbal medicine intervention is a safe and effective alternative medicine for the treatment of asthma.

In recent years, many studies that focus on TCM suggest that recurrent asthma has important links with wind (*Feng*), phlegm (*Tan*), blood stasis (*Yu*), and deficiency (*Xu*) [18]. In the Qing dynasty in China, a prescription of the *Yang He* (YH) formula was developed, and was commonly used for the treatment of the *Yin* and *Han* syndromes [19]. It functions in reinforcing deficiency (*Fu Zheng*), as well as dispelling wind (*Qu Feng*), removing blood stasis (*Hua Yu*), and eliminating phlegm (*Qu Tan*). Based on the empirical prescription, ingredients such as *Asari Radix Rhizoma* (*Xi Xin*) and *Codonopsis Radix* (*Dang Shen*) were added to improve the YH formula, thereby generating the *Jia Wei Yang He* (JWYH) formula [20, 21]. JWYH strengthens the function of reinforcing deficiency and resolving phlegm, and has been widely used for the treatment of chronic asthma. Our preliminary data show that the JWYH formula significantly improves cough and sputum symptoms, reduces the number of asthma episodes, and improves serum interferon (IFN)-γ levels (data not shown).

In several epidemiological studies, an association is reported between respiratory infections and the pathogenesis of bronchial asthma [22]. In addition, samples collected by bronchoscopy or induced sputum indicate a relation between asthma and microbial infection of the airway pathogens, including common bacteria such as *Proteobacteria*, *Bacteroidetes*, and *Firmicutes* [23]. Based on these previous observations, we hypothesize that TCM treatment modulates airway micro-organisms, and changes both airway microbiome composition and function, thereby impacting the symptoms of asthma. To the best of our knowledge, changes in microbiome composition in the airways of asthma patients with TCM treatment have not yet been fully characterized.

In previous studies, it is shown that JWYH therapy improves the symptoms of asthma and prevents the condition from worsening [18]. However, no data are available from large-scale randomized controlled trials (RCTs) on the efficacy of JWYH and its adverse effects. Therefore, we aim to perform the first double-blinded, randomized, placebo-controlled trial to determine the effectiveness and safety of JWYH treatment for persistent asthma.

## Methods/design

### Study design

**Fig. 1:**
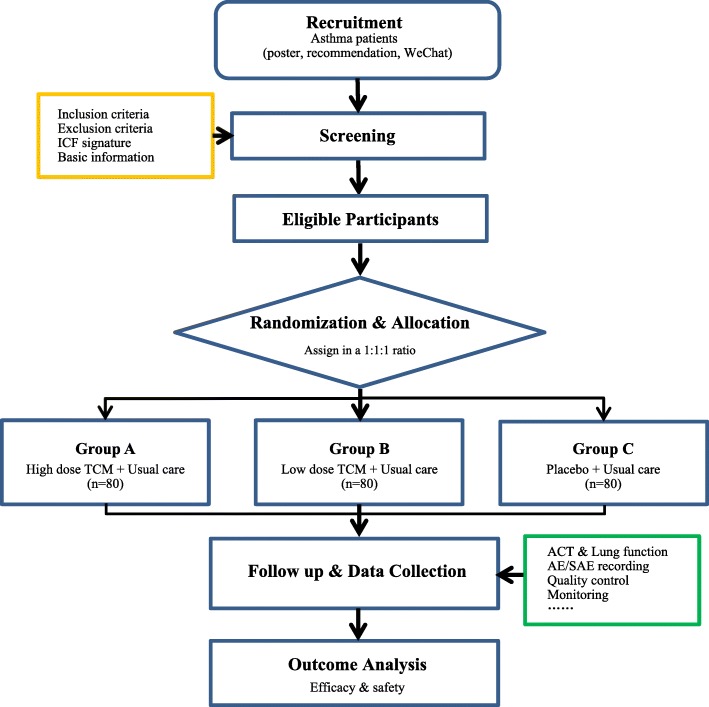
Illustration of study design for clinical studies

This study is a multicenter, double-blinded, placebo-controlled, randomized, 4-week treatment clinical trial. The course of the study consists of three periods: a 1- to 3-day screening period, a 4-week primary treatment period, and a 3-month follow-up period. Follow-up assessments will be conducted at monthly clinic visits and will last for 3 months (Fig. 1). In this study, the clinical efficacy of JWYH is assessed in the treatment of asthma, identifying associations between the airway microbiome and asthma, and to examine if changes in the airway microbiome occur for asthma patients who receive daily TCM treatment.

### Organization

The principal investigator (PI) is responsible for the overall project and for organizing Steering Committee meetings. PIs of sub-center departments are responsible for gathering experts to carry out the project. An independent Steering Committee will be responsible for the overall safety of participants, coordination of study meetings, feedback progress of recruitment or follow-up of participants, supervising the study, and overseeing quality control studies. The coordinating center is responsible for communicating protocol modifications and for providing materials.

### Setting

This clinical trial will be conducted in eight clinical centers in Shanghai: Longhua Hospital affiliated Shanghai University of TCM, Fengxian District TCM Hospital, Jing’an Centralized Hospital, Pudong Hospital, Eighth People’s Hospital of Shanghai, Shanghai TCM-Integrated Hospital, Xuhui Centeralized Hospital, and the Fudan University affiliated Zhongshan Hospital. Prior to the trial, all personnel are trained in the Longhua Hospital to ensure that the multicenter physicians and staff involved in the trial fully understand all aspects of the trial.

### Aims

#### Primary outcome

The primary outcome for the assessment of effectiveness includes mean differences in the Asthma Control Test (ACT) score between JWYH-treated and placebo-treated groups at week 4 from baseline. The ACT involves a five-question, patient-based tool for identifying patients with poorly controlled asthma. This score shows to be a reliable and valid measure that has been used to accurately evaluate changes in asthma control in different patient populations over time [24–27].

#### Secondary outcomes

Secondary outcomes included:

- ACT score at week 8
- Asthma exacerbation rate (AER) at week 8
- Percentage changes in FEV_1_ from baseline at week-4 and week-8 follow-up visits
- Percentage changes in FVC from baseline at week-4 and week-8 follow-up visits
- Changes of fractional exhaled nitric oxide (FeNO) at week-4 and week-8 follow-up visits
- Daytime and nighttime peak expiratory flow (PEF) at week-4 and week-8 follow-up visits

### Exploratory outcome

ACT score at week 16

Microbial diversity: We hypothesize that airway microbiota is different among TCM treatment and control groups regarding changes in microbial diversity. The composition and structure of the microbiome in induced sputum will be tracked using high-throughput *16SrRNA*-based gene analysis. Diversity index of evenness, richness, and abundance will be calculated to summarize and compare microbiomes at the operational taxonomic unit (OTU) level.

### Participants

Patients who meet the inclusion criteria and are not excluded by the exclusion criteria (Table 1) will be invited to participate in this study. Every year, the collaborating centers have many asthmatic outpatients and inpatients, which significantly facilitates subject recruitment. Prior to the start of the study, patients are informed of all aspects of the study. The PIs and/or research nurse explain the study in detail, answer questions, ensure that informed consent will be given by each patient, and that appropriate signatures are obtained prior to the start of the study.

**Table 1:** Inclusion and exclusion criteria

Inclusion criteria	
1. Patient diagnosed with chronic persistent asthma	
2. Annual uncontrollable time ≥ 3 months	
3. Patients who have given written informed consent	
Exclusion criteria	
1. History of upper upper/lower respiratory infection in the previous 1 month	
2. History of long-term controller medication use for asthma (orally or intravenously administered corticosteroid therapy) within the preceding 1 month	
3. History of antibiotic use in the previous 1 month	
4. History of life-threatening asthma	
5. History of chronic lung diseases	
6. History of serious disease of the heart and cerebrovascular disease	
7. History of severe liver or renal dysfunction or disease	
8. History of severe disease in the hematopoietic system	
9. History of immunodeficiency (including, but not limited to, HIV-positive detection)	
10. History of any other condition (such as known drug or alcohol abuse or psychiatric disorder)	
11. Are currently enrolled in, or discontinued within the last 30 days from, a clinical trial involving an investigational product or non-approved use of a drug or device	
12. History of allergies to the component of the investigational drugs	
13. Smoking within the past year	
14. Contraindication to induced sputum collection method on history or examination	
15. Any serious medical condition which, in the opinion of the investigator, would pose a significant risk to the patient or interfere with the interpretation of safety, efficacy, or pharmacodynamic data	

After the informed consent form (ICF) is signed and dated, a patient is considered “eligible” to enter the trial, is assigned a patient number, and is randomized to one of three treatment arms.

The two primary resources for identifying and recruiting potential subjects are as follows:

1. Participants are recruited from the outpatient departments of the eight clinical centers in Shanghai
2. We advertise in Longhua official accounts on WeChat and distribute printed posters in public clinics and nearby communities

We will continue to provide additional health care or compensation for participants’ health care needs that arise as a direct consequence of trial participation.

### Treatments administered

Patients are randomly assigned to groups to either receive a high-dose JWYH by oral administration, a low-dose JWYH by oral administration, or a placebo twice daily for 4 weeks (28 days). After baseline assessment, all participants will maintain or receive background therapy with ICS or a β_2_-adrenoceptor agonist as usual care.

Participants in the high-JWYH-formula group receive 18.5 g JWYH granules twice daily for 4 weeks, whereas participants in the low-JWYH-formula group receive 9.25 g of JWYH granules twice daily for 4 weeks. Patients in the placebo group are given placebo granules (9.25 g twice daily for 4 weeks). Drugs are identical in appearance, shape, size, and packaging.

Whole ingredients of the intervention formula include *Rehmanniae Radix Praeparata* (*Shu Di Huang*) 15 g, *Cinnamomi Cortex* (*Rou Gui*) 3 g, *Ephedrae Herba* (*Ma Huang*) 4.5 g, *Cervi Cornus Colla* (*Lu Jiao Jiao*) 3 g, *Sinapis Semen* (*Bai Jie Zi*) 6 g, *Zingiberis Rhizoma Preparatum* (*Pao Jiang*) 6 g, *Clycyrrhizae Radix Et Rhizoma* (*Gan Cao*) 6 g, *Asari Radix Rhizoma* (*Xi Xin*) 3 g, and *Codonopsis Radix* (*Dang Shen*) 9 g. All herbal granules are produced by the Sichuan Green Pharmaceutical Technology Development Limited by Share Ltd. in Sichuan, China. The dosage for all three groups includes one pack of granules that needs to be soaked in 100 mL of boiling water, taken orally twice daily, after breakfast and supper.

At each study visit, adherence to intervention is monitored and participants are asked to return all study containers with any unused packs of granules, including all empty containers. Once the patient is randomized, study sites will take every reasonable effort to follow the patient for the entire course of the study. All examination and transportation costs are covered and the results of symptoms and physical examinations will be explained at every visit. Prior to every visit, messages will be sent through WeChat or by phone to remind patients of the upcoming data collection.

Patients who discontinue the assigned treatment based on serious adverse events (SAEs), or poor compliance (more than 14 days without taking medicine), or in cases when the attending physician requests that the patient should undergo orally or intravenously administered corticosteroid therapy because symptoms worsen after screening.

### Randomization and blinding

For each group, a block randomization list is provided, which is generated prior to initiation of the study using a random seed number via SAS PROC PLAN software (SAS Inc., Cary, NC, USA). Randomization occurs after screening and baseline assessments, and patients will be randomized in a 1:1:1 fashion to receive one of three treatments. Participants will be randomized using web-based randomization service, which is an online, central randomization service provided by Longhua Clinical Trials Support Unit in Shanghai, China. This service will not release the randomization sequence until the interventions are assigned.

All investigators involved will be blinded to group assignment. An independent data analyst and the research nurse who assigns subjects will not be blinded to treatment status and will not take part in any other part of this trial.

If, after the first administration, any clinically significant adverse event is potentially related to JWYH treatment, the study physician will re-evaluate the participant and local PI or delegate will decide whether a non-blinded procedure is necessary. If non-blinding is considered required, the actual allocation information will be provided to the physician.

### Data collection and management

**Fig. 2:**
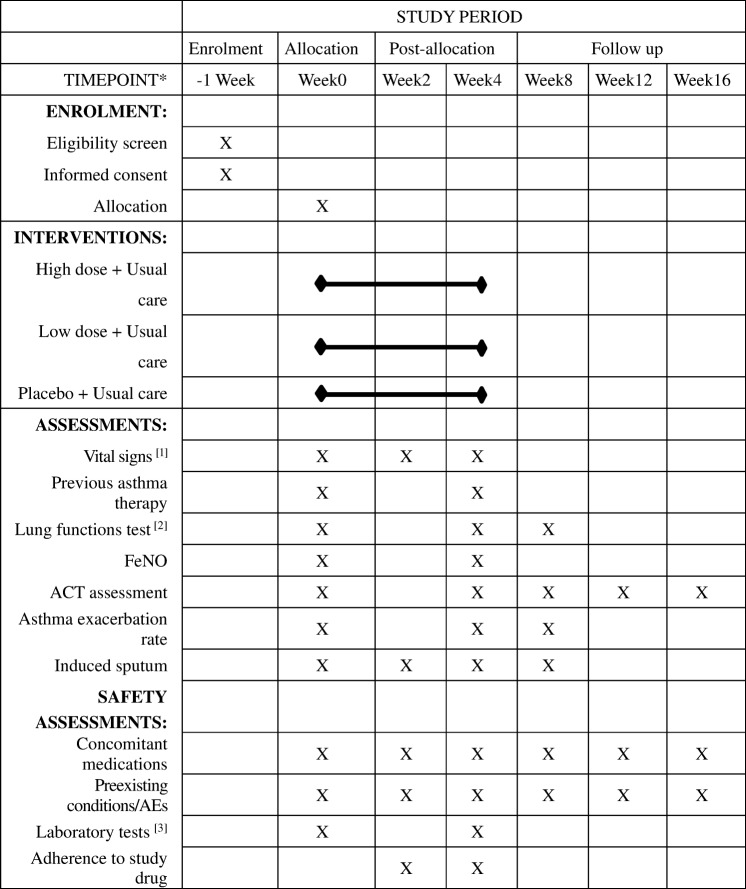
Schedule for enrollment, intervention, and assessment. *List specific time points in this row. [1] Vital signs: height, weight, body temperature, blood pressure, and heart rate. [2] Lung function tests: *FEV*_*1*_ forced expiratory volume in 1 s, *FEV*_*1*_*/FVC*, *FVC* forced vital capacity, *IC* inspiratory capacity, *TLC* total lung capacity. [3] Laboratory tests: blood, urine, feces, electrocardiogram, kidney and liver function

After screening and completing a set of questionnaires and evaluations, participants will attend two assessment visits and three follow-up visits (Fig. 2). Demographic information (date of birth, gender, etc.) and medical condition (medical history, concomitant medication, etc.) are recorded.

### Lung function

Lung function will be assessed by FEV_1_, FVC, and PEF. Lung function tests are conducted at baseline and every 4 weeks with standardized equipment (Erich Jaeger UK Ltd., Market Harborough, UK Jaeger Master-Screen, Germany) and standardized procedures recommended by the American Thoracic Society (ATS) [28]. After randomization, a peak flow meter and asthma diary will be given to each patient, who will be trained to use the equipment to record their PEF daily. FeNO will be conducted according to ATS standards [29].

### Collection of sputum

At the time of randomization, samples of induced sputum will be collected from subjects, as well as at 2 weeks, 4 weeks, and 8 weeks after enrollment. From each subject, sputum will be induced by inhalation of a nebulized solution of 3% saline. To minimize oral contamination, patients rinse their mouth and clean their nasal passages before sputum induction. Sputum samples are collected into sterile cups, and the entire procedure lasts about 15 min. Sputum plugs are selected and differential cell counting is performed. An equal weight of dithiothreitol is added to the sputum plug to dissolve mucus. All samples will be stored at − 80 °C prior to bacterial deoxyribonucleic acid (DNA) extraction.

### Data management

All records will be collected in Case Report Form (CRF) files, and data input will be employed using a double independent entry to ensure data accuracy. Once verification is completed, data will be securely stored.

Original study forms will be entered and kept on file at the participating site. All forms are sent to the coordinating center, and data are entered into EpiData 3.1 software by two study nurses (independent double-data entry by two individuals). Data errors and inconsistencies will be checked during the entry process. Files are stored in a secure and accessible place and manner. Security measures will be regulated by a user identification code and password. Monthly backups are stored on CD media. Finally, the database will be locked and analyzed under the agreement and confirmed review of the PI.

The PI will be given access to the cleaned data sets. Site investigators will have direct access to the data sets from their own sites, and will have access to the data on other sites on request.

### Safety

In China, *Yang He* decoction has been used for hundreds of years, and the dosage of JWYH used in this study is within the recommended range based on the *People’s Republic of China Pharmacopeia* (2015 edition). Due to the long-time clinical use and preliminary studies of JWYH from January 2017 to April 2017, no adverse effects were observed during the treatment and follow-up period. Moreover, we will employ a series of measures, including subjective description and laboratory tests, that especially focus on gastrointestinal intolerance, heart, liver, and kidney damage to assess the safety of JWYH from the time of enrollment through the follow-up period. During each visit, patients will be asked about any adverse effects during the study period. In addition, laboratory tests will be conducted on blood, urine, feces, electrocardiogram, kidneys and liver function prior to and after treatment. If adverse events are observed, we are prepared to immediately provide appropriate examination and treatment.

Any adverse event that occurs to a subject will be recorded in the CRF regardless of relationship to the study intervention. In case of any serious adverse events (SAEs), the intervention will be immediately stopped and a detailed description of the time, severity, relationship with the drug and the measures taken of the SAE based on standard operation procedures (SOPs) of the China Food and Drug Administration (CFDA) will be given. In addition, SAEs will be reported to the PI, Steering Committee, Ethics Committee, and the sponsor within 24 h.

### Sample size

Sample size calculations are based on the primary endpoint (ACT score at 4 weeks). At the 5% significance level, a total of 64 patients per group is required to achieve 80% power and to determine an increase of 2 points in the ACT score between the high-dose TCM plus usual care group and the placebo plus usual care group, assuming that the standard deviation of the groups is equal to 4 [30]. If the previous hypothesis is rejected, then the difference of 1.5 points in ACT score between the low-dose TCM plus usual care group and the placebo plus usual care group at the 5% significance level is tested. With an estimated dropout rate of 20%, a total of 240 patients is enrolled. If the initial hypothesis is not rejected, subsequent comparison is considered exploratory, and no conclusion is made [31, 32].

### Statistical methods

The modified intent-to-treat (mITT) population is defined as randomized patients who receive at least one dose of randomized treatment with at least one post-randomization ACT value, classified according to their assigned treatment. The mITT analysis set is used for efficacy and exploratory analyses. Last observation carried forward (LOCF) imputation is used for missing data in the mITT population. A per-protocol (PP) population is defined as randomized patients without major protocol violations. The PP analysis set is used to repeat the efficacy analysis and to assess the robustness of the results. The safety population is defined as all patients who receive at least one dose of randomized treatment and for whom any post-dose data are available, classified according to the treatment received. The safety population will be used for the analyses of all safety measures.

For the primary outcome, changes in ACT scores from baseline to week 4 will be analyzed by means of analysis of covariance (ANCOVA). Treatment and center interactions will be examined, and, when an interaction does not exist, the ANCOVA model includes treatment and baseline ACT total score as explanatory variables. Otherwise, ANCOVA analysis will include the baseline ACT total score as covariate, treatment as a fixed effect, and the center as a random effect. Secondary outcomes (ACT at 8 weeks, FEV_1_, and FVC) are assessed in a similar fashion. AER will be analyzed using a *t* test or Wilcoxon rank-sum test as appropriate, and the Holm-Bonferroni method is used to control type I errors if necessary.

A mixed-model repeated measures (MMRM) analysis will be used to assess PEF changes. Comparisons will be based upon the contrast between treatments at week 4 (day 28) and week 8 (day 56). Model-based point estimates, 95% confidence intervals, and *p* values will be calculated. A *p* value < 0.05 is considered statistically significant.

For exploratory analysis, MMRM analysis uses all available ACT total score through the week 16 (day 112) visit, and comparisons will be based upon the contrast between treatments at week 16 (day 112). Model-based point estimates, 95% CIs, and *p* values are calculated.

Any adverse event will be recorded in the CRF regardless of relationship to the study intervention. Safety endpoints will be analyzed using summary statistics (frequency, count, percentage).

Where appropriate, subgroup analyses, will be provided for effectiveness and safety endpoints, and will be based on baseline disease characteristics.

Data are analyzed using SAS 9.4 software (SAS Institute Inc., Cary, NC, USA). Unless otherwise noted, all treatment effects are employed at a two-sided α level of 0.05 and confidence intervals (CIs) will be calculated at 95%, two-sided.

For exploratory analysis of the airway microbiome, alpha diversity will be estimated as the number of observed OTUs and by the Chao1 Index, Simpson Index, Shannon Index, and phylogenetic alpha diversity (PD). UniFrac beta-diversity metrics will be compared between treatment groups using the PERMANOVA test from R’s vegan library [33]. In addition, UniFrac measures the phylogenetic distance between two samples as the fraction of the branch length of the phylogenetic tree, and are widely used to determine whether communities are significantly different [34]. Community dissimilarities will be estimated using principal coordinates analysis (PCoA). OTU abundance differences will be analyzed by assuming rarefied (Fisher’s exact test, respectively) and non-rarefied (metagenomeSeq zero-inflated Gaussian [35]) data. Statistical significance is determined by permutation tests using 10,000 permutations. In addition, core microbiome analyses will be performed to identify resident bacteria, after applying Benjamini-Hochberg FDR adjustments for multiple comparisons.

### Major protocol deviation

The major protocol deviation may affect the risk/benefit ratio of the study, the rights, safety or welfare of the participants, or the integrity of the study. The major protocol deviation is defined as failure to obtain informed consent, omit the research procedures required by approved protocols, drug distribution/dose errors, and eliminating the necessary deviations necessary for the obvious immediate harm to participants.

### Quality control and monitoring

There will be no interim analyses. During the study, an independent Steering Committee will review and oversee all the source documents and CRFs. The PI and quality assurance personnel will audit the data and trial conducted by personal visits or telephone (monthly as needed) to ensure compliance with the protocol and the quality of the data at every site. The essential documents (consent information, enrollment, protocol deviations, number and proportion of missed visits and losses to follow-up) will be monitored and checked for accuracy and completeness by the monitors.

### Data sharing and dissemination plan

For sharing purposes, data will be available to outside investigators at the end of the trial. The findings of this study will be published in peer-reviewed journals and presented at conferences. The study results will be released to the participating physicians and patients.

## Discussion

Chinese medicine, especially *Wen Yang* herbs, which are similar to JWYH, can enhance human cell immunity, including innate immune function [36], thereby helping the body to improve its immune function and combat diseases. JWYH is composed of *Asari Radix Rhizoma*, *Codonopsis Radix,* and YH formula. Clinical studies demonstrate that YH relieved asthma symptoms to a certain extent and significantly decreased high serum levels of serum endothelin (ET), vascular endothelial growth factor (VEGF), and basic fibroblast growth factor (b-FGF), improving airway remodeling [18]. These effects may be due to its ingredients of *Cervi Cornus Colla*, *Ephedrae Herba*, and *Sinapis Semen*. *Ephedrae Herba* may ameliorate the progression of asthma, and was further investigated for potential use as a therapy for patients with allergic asthma [37], whereas oral intake of *Cervi Cornus Colla* may be an effective way of alleviating asthmatic symptoms in humans [38].

In recent years, three Chinese evidence-based trials reported that YH had significant beneficial effects on asthma treatment [39–41]. However, none of these trials are double blinded. Furthermore, the outcome measurements, such as PEF and wheezing, can be easily affected by environment factors and individual patient characteristics. Therefore, these outcomes cannot completely reflect improvement of asthma symptoms and quality of life. In our study, we employ validated and objective tools, such as the ACT score and FEV_1_ as outcome measurements. These measurements improve the reliability and generalizability of the results.

Microbial communities inhabiting in the human body have been shown to be associated with the health status of the host [42–45]. Based on the *16SrRNA* gene-sequencing technique, which does not depend on culture, the bacterial 16S ribosomal subunit variable region has been sequenced and evolutionary relationships among millions of bacteria were defined [46–48]. In recent years, several studies have indicated a significant correlation between asthma symptoms and specific microbial species in the lower airways [49–51]. In addition, studies of induced sputum microbiome at the *16SrRNA* gene level reveal a different composition of bacterial microbiota between asthmatic patients and healthy individuals [50, 52]. In this clinical trial, we will investigate changes in microbial composition and examine whether any differences are the consequence or indication of asthma.

This double-blinded RCT will provide high-quality evidence in evaluating the efficacy and safety of JWYH as an adjunctive treatment to reduce the symptoms and improve the quality of life in persistent-asthma patients. The results should contribute to decision-making in the process of asthma treatment and management and should provide significant information that can be incorporated into future guidelines.

## Trial status

In this study, we describe the protocol of the trial, version 1.3, 15 December 2017. This is a current ongoing trial, which actively recruits subjects. We anticipate patient recruitment to finish by 1 December 2020, and will report the final study results the following year (Additional file 1).

## Additional file

Additional file 1: Standard Protocol Items: Recommendations for Interventional Trials (SPIRIT) 2013 Checklist: recommended items to address in a clinical trial protocol and related documents. (DOC 125 kb)

## Abbreviations

ACT: Asthma Control Test
AER: Asthma exacerbation rate
ANCOVA: Analysis of covariance
CAM: Complementary and alternative medicine
CIs: Confidence intervals
FEV_1_: Forced expiratory volume in 1 s
FVC: Forced vital capacity
GINA: Global Initiative on Asthma
ICF: Informed consent form
JWYH: *Jia Wei Yang He*
LOCF: Last observation carried forward
mITT: Modified intent-to-treat
MMRM: Mixed-model repeated measures
OTUs: Operational taxonomic units
PCoA: Principal coordinates analysis
PD: Phylogenetic alpha diversity
PEF: Peak expiratory flow
PI: Principal investigator
PP: Per protocol
RCTs: Randomized controlled trials
SAEs: Serious adverse events
SOPs: Standard operation procedures
TCM: Traditional Chinese medicine
